# Low-cost evaluation and real-time feedback of static and dynamic weight bearing asymmetry in patients undergoing in-patient physiotherapy rehabilitation for neurological conditions

**DOI:** 10.1186/1743-0003-10-74

**Published:** 2013-07-12

**Authors:** Joanna Foo, Kade Paterson, Gavin Williams, Ross Clark

**Affiliations:** 1 Centre of Physical Activity Across the Lifespan, School of Exercise Science, Australian Catholic University, Fitzroy, VIC, Australia; 2 Department of Physiotherapy, Faculty of Medicine, Dentistry and Health Sciences, The University of Melbourne, Parkville, VIC, Australia; 3 Physiotherapy department, Epworth hospital, Richmond, VIC, Australia

**Keywords:** Balance, Neurology, Postural control, Weight distribution, Brain injury

## Abstract

**Background:**

Weight bearing asymmetry is common in patients with neurological conditions, and recent advances in gaming technology have produced force platforms that are suitable for use in a clinical setting. The aim of this research is to determine whether commercially-available Wii Balance Boards with customized software providing real-time feedback could be used in a clinical setting to evaluate and improve weight-bearing asymmetry in people with various neurological conditions.

**Methods:**

Twenty participants (age = 43.25 ± 19.37 years) receiving physiotherapy as a result of a neurological condition performed three trials each of two tasks (static standing and sit-to-stand) with and without visual feedback. Vertical forces were measured using available Wii Balance Boards coupled with customized software that displayed visual feedback in real-time. Primary outcome measures included weight-bearing asymmetry as a percentage of body mass, peak force symmetry index, and a visual analogue scale score rating self-perceived level of asymmetry.

**Results:**

Weight-bearing asymmetry during the static balance task was significantly reduced (Z = −2.912, *p* = 0.004, ES = 0.65) with visual feedback. There was no significant difference (Z = −0.336, *p* = 0.737) with visual feedback for the dynamic task, however subgroup analysis indicated that those with higher weight-bearing asymmetry responded the most to feedback. Correlation analysis revealed little or no relationship between participant perception of weight-bearing asymmetry and the results for the static or dynamic balance task (Spearman’s rho: ρ = 0.138, *p* = 0.561 and ρ = 0.018, ρ =0.940 respectively).

**Conclusions:**

These findings suggest that weight-bearing asymmetry can be reduced during static tasks in patients with neurological conditions using inexpensive commercially-available Wii Balance Boards coupled with customized visual feedback software. Further research is needed to determine whether real-time visual feedback is appropriate for reducing dynamic weight-bearing asymmetry, whether improvements result in improved physical function, and how cognitive and physical impairments influence the patient’s ability to respond to treatment.

## Background

Greater weight bearing asymmetry (WBA) is associated with increased postural instability [1]. In neurological populations, high levels of WBA are commonly reported [2] and some measures of dynamic asymmetry have been associated with an increased falls risk in older adults [3-5]. This in turn leads to an increased risk of further injury [6], institutionalisation [7] and/or death [8]. Although controversy exists regarding whether WBA is due to the constraints of the neurological pathology or is a central nervous system adaptation [1], improving symmetry remains a goal for patients with an acquired brain injury or other neurological condition due to these adverse consequences [9].

The evaluation of WBA typically occurs in a laboratory using multiple force platforms [10] and is often combined with visual or auditory biofeedback of inter-limb force distribution [11,12]. Whilst research has shown this equipment can be effective at improving symmetry and balance in neurological populations [13], it is also expensive, difficult to transport and requires expert technical knowledge, thereby limiting its use in a clinical setting. In contrast, visual or observational assessments are commonly employed by clinicians as they are quick, inexpensive and easily accessed and applied, however the reliability and validity of these techniques are often poor [14], particularly during dynamic tasks [12,15]. Finally, although some previous studies have used digital scales to evaluate standing balance in stroke patients [16], these instruments are not suitable for assessing balance during dynamic activities

Recent research has demonstrated that inexpensive commercially-available balance boards can provide an accurate and reliable measure of WBA [17-19]. The low cost and portability of these systems could lead to them being used more frequently in a clinical setting, as both an assessment and rehabilitation tool. Indeed, other work has shown this equipment may be used in combination with customized training software that incorporates force data acquisition and real-time feedback to improve performance on tests of static and dynamic balance [20]. Such feedback is important given patients with neurological disorders have been reported to be unable to perceive their level of asymmetry [12]. The aim of this study was to use two balance boards with customized software to rapidly evaluate WBA during static and dynamic balance tasks in people with a neurological condition, and to determine whether customised software displaying real-time feedback could be used to improve WBA. An additional aim was to evaluate whether self-perception of asymmetry during balance tasks is related to actual WBA. It is hypothesized that patients with a neurological condition will have WBA during static and dynamic balance tasks, which will improve with real-time visual feedback. It is also hypothesized that there will be a weak relationship between self-perceived and actual WBA.

## Method

### Participants

Twenty people with various neurological conditions, aged between 18 and 55 years (age = 43.25 ± 19.37 years, body mass = 81.4 ± 19.54 kg, height = 171.5 ± 7.77 cm, 10 m walk time = 14.54 ± 10.02 sec), volunteered to participate in this study. A heterogeneous sample of neurological patients was chosen to reflect a typical rehabilitation setting. Participants were recruited from the neurological rehabilitation unit at the [removed]. Patients were invited to participate if they were receiving physiotherapy for mobility limitations as a result of a neurological condition, and were excluded if they were unwilling or unable to provide informed consent, had severe cognitive or behavioural problems that would prevent assessment, or had a pre-existing condition which may impact on their gait performance or ability to process visual feedback. Eleven participants had sustained a traumatic brain injury, three had tumours of the central nervous system, two had a stroke, two had cerebral palsy, one had sustained a spinal cord injury and one had a hypoxic brain injury. Six participants required a gait aid, of which four used forearm crutches and two used a walking frame. Gait aids were not used during testing as the assessment tasks did not require locomotion, and use of a gait aid during standing would distribute force away from the testing equipment and therefore reduce the quality of the results. The average time post injury (excluding cerebral palsy) was 23.3 ± 33.6 months. The study was approved by the [removed] Human Research Ethics Committee and the [removed] Hospital Human Research Ethics Committee.

### Procedure

Two Nintendo Wii Balance Boards™ (NWBB) were used to measure WBA during each balance task, using a software system and data collection protocol similar to that described previously [19]. Briefly, the individually calibrated force sensor data from each NWBB was transferred to a computer via a Bluetooth connection, synchronized and interpolated to 40Hz, and then filtered using a Wavelet-based filter with a low pass cut-off frequency of 10 Hz. The reliability of a similar dual NWBB based system for evaluating WBA has previously been established (within-device ICC_2,1_ range = 0.66–0.94, between-device comparison with a force plate ICC_2,1_ range 0.77-0.89) [17,19].

Participants were required to complete a series of two static and dynamic balance tasks chosen to represent activities of daily living. The first was a static standing balance task that was completed under two conditions. In the first condition, the participant was required to stand as still and as evenly as possible in a comfortable position with one foot on each NWBB. For the second condition, participants performed the same task with real-time visual feedback of their weight distribution and were instructed to use this feedback in an attempt to reduce WBA. Data for each condition were collected over three 30 second trials with 30 seconds rest between each trial.

The second task evaluated dynamic WBA whilst performing a sit-to-stand movement, and this was also completed under two conditions. In the first condition, participants were seated on an armless and backless chair with each foot positioned 10 to 15 cm apart parallel on the centre of each NWBB, and in approximately 10 degrees of dorsiflexion. The chair height was then adjusted to the height of the participant’s knee and at an angle of 100 – 105 degrees of knee flexion [21,22]. Participants were then instructed to rise from a seated position to a standing position, pause, and then return back to a seated position, with three repetitions completed at the participant’s own pace. Three trials each of three sit-to-stand/stand-to-sit movements were completed with each trial performed when the participant had recovered from the previous trial. The task was then repeated with visual feedback regarding WBA provided to the participant whilst they attempted to use this feedback to reduce their WBA. Prior to testing, participants were instructed to cross their arms on their chest to ensure their hands were not used to assist in the standing or sitting movements. For each of the tasks in each of the visual feedback conditions participants were provided with a minimum of one practice trial to ensure familiarity with the visual feedback software.

Task order was not randomised for two reasons. Firstly, as the dynamic task was more difficult, it may have been strenuous for some participants. Therefore the sit-to-stand task was performed second to ensure participants were not fatigued during the static tasks. If some participants performed the dynamic task first, it is possible that their postural sway may have increased due to fatigue, leading to increased WBA [23]. Additionally, the visual feedback conditions were also not randomised to eliminate any learning effect. That is, if a participant were to receive real-time visual feedback on their weight distribution in their first trial, they may be able to adjust their limb loading in subsequent trials where no real-time visual feedback is provided.

In between the two visual feedback conditions, data were also collected on the participant’s self-perception of weight bearing symmetry during the no visual feedback condition using a 100 mm visual analogue scale (VAS). This protocol replicated the methodology of Briere et al. [12], and was used to ascertain self perception of WBA during the specific task assessed. Briefly, the participants were asked to indicate on the VAS where they felt their weight was distributed during the task, where the left, right and middle of the scale indicated their weight was 100% on the left, 100% on the right or equally distributed (50:50%) respectively.

### Data analysis

**Figure 1:**
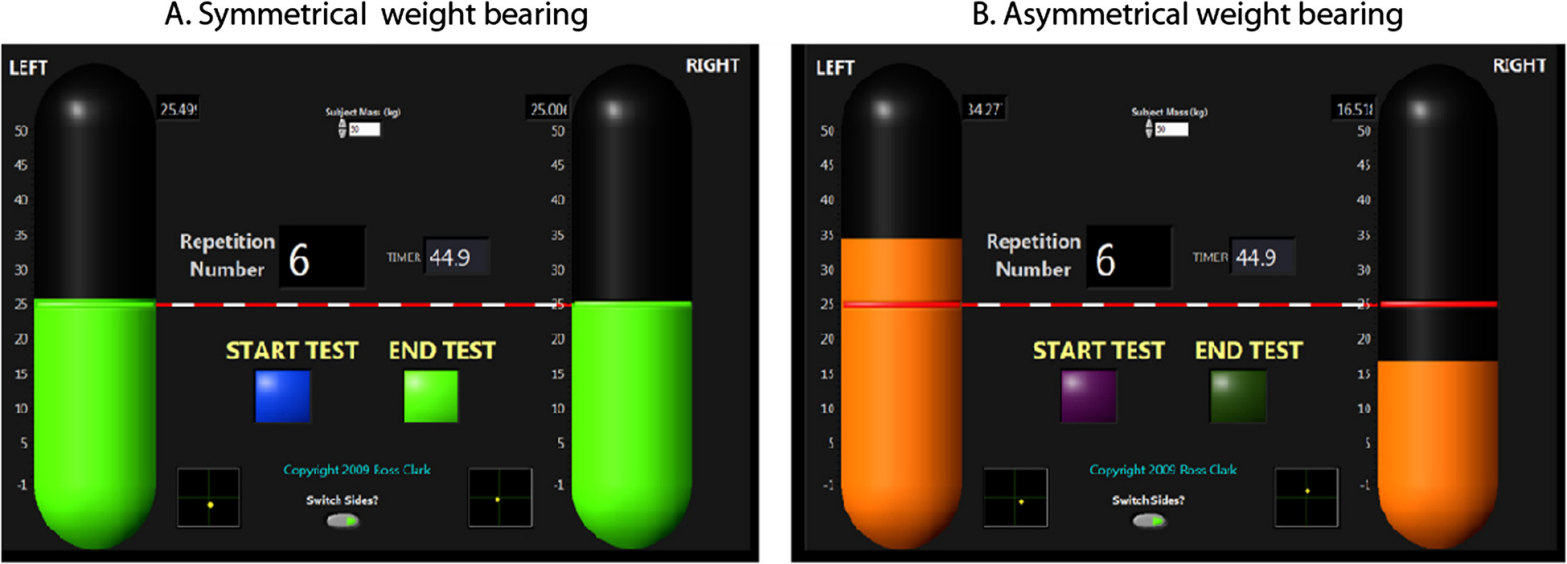
**Real-time visual feedback program.** Figure **A** is an example of asymmetrical loading, with uneven vertical bars. These values fall outside the symmetrical load range, and therefore would be displayed in the color orange. Figure **B** is an example of symmetrical loading, and the vertical bars would be displayed in the color green.

When required, visual feedback was relayed to the participant using custom made software (Labview, version 8.5). This information was displayed on a large computer screen positioned approximately two meters directly in front of the participant. Weight distribution for each limb was displayed as left and right vertical bars, which fluctuated according to how much load was on each NWBB. The bars were coloured green and positioned at an even height when weight distribution was symmetrical and turned orange and positioned at an uneven height when weight bearing was asymmetrical (see Figure 1). A value of greater than 5% of half of the participant’s body weight on each limb was chosen to indicate asymmetry as this value represents a typical WBA that is unlikely to be attributed to random weight distribution fluctuation observed in quiet stance [18].

The WBA expressed as a percentage of body mass was used to provide a measure of overall WBA amongst both tasks [19]. The peak force symmetry index (pfSI) was used as a measure of asymmetry in limb loading during the dynamic balance task. It is calculated by subtracting the peak force distributed through the right limb from the peak force distributed through the left limb and dividing this value by half of the participant’s body mass. This value was then multiplied by 100 in order to express it as a percentage, with the absolute value used for analysis. This provides an indication of how symmetrical the participant is from the beginning of the sit to stand movement through to the point where peak force has been reached [24]. Self-perception of WBA was measured using a 100 mm VAS [12]. It was calculated by measuring (mm) the participants’ indication of where their weight was distributed during the first visual feedback condition for both the static balance task and the dynamic balance task. A mark to the left of the middle point on the VAS was considered a negative score (cm) and a mark to the right of the middle point on the VAS was considered a positive score (cm).

### Statistical analysis

Normality assessment using the Shapiro-Wilk test revealed that the outcome measures were not normally distributed, therefore non-parametric statistics were used. The effectiveness of visual feedback on each task was assessed by comparing the differences between the no visual feedback and visual feedback conditions using a Wilcoxon Signed Rank Test and associated effect size (ES) statistics were calculated for any significant findings. To determine the relationship between the initial asymmetry level and the response to visual feedback, linear regression was performed using the results of the no feedback condition and the change in asymmetry from the no visual feedback to feedback conditions. To determine the non-linearity of this relationship, a locally weighted scatterplot smoothing line was also fitted to these data. These analyses were performed online using the linear regression and LOWESS functions in the statistical program R [25]. Evaluation of participants’ perception of their weight-bearing symmetry was performed by correlating the data collected on the VAS with the results of the no visual feedback condition, using a Spearman’s Rank Order Correlation analysis. For these correlation analyses the values for weight-bearing asymmetry and pfSI were assessed prior to converting them to absolute values, with additional load on the left limb resulting in a negative number. All comparisons were made with an alpha level set at *p* < 0.05.

## Results

All participants were able to complete the tasks with no adverse events. The results of the entire sample for the measures of WBA in each of the two feedback conditions are represented in Table 1. Real-time visual feedback was found to significantly reduce WBA during the static standing balance condition (Z = −2.800, *p* = 0.005, ES = 0.63). In contrast, no significant differences in the pfSI (Z = 0.336, *p* = 0.737) were found between visual feedback conditions during the dynamic sit-to-stand task.

**Table 1:** Median (inter-quartile range) weight bearing asymmetry results during the static (quiet standing) and dynamic (sit-to-stand) trials performed with and without feedback

**Condition**	**No Feedback**	**Feedback**	**Z-score**	***p***
Static Trial (%BM)	6.30(1.79–10.82)	2.46(0.27–4.66)	2.80	0.004
Dynamic Trial (pfSI)	14.99(6.57–23.41)	19.82(10.09–29.55)	-0.336	0.737

Abbreviations: BM, body mass; pfSI, peak force symmetry index.

**Figure 2:**
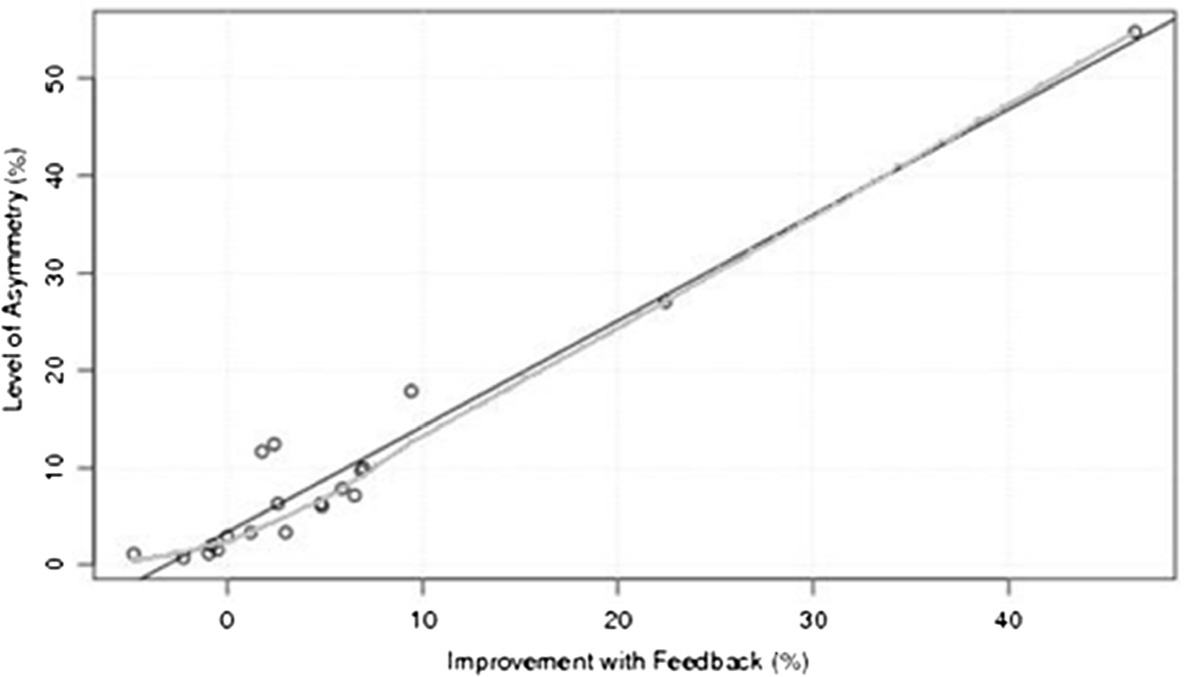
**Comparison of the improvement of weight bearing asymmetry during the visual feedback condition with the initial level of weight bearing asymmetry for the static balance task.** The black, straight line represents the linear fit (F = 323, *p* < 0.001, R^2^ = 0.947), the red, irregular line is indicative of the locally weighted scatterplot smoothing line.

The linear regression analysis of the initial level of WBA recorded in the no feedback trials and the response to visual feedback revealed a significant and strong positive relationship (F = 323, *p* < 0.001, R^2^ = 0.947) during the static balance task, with higher initial levels of WBA associated with a greater response to feedback (Figure 2). The shape of the locally weighted scatterplot smoothing line suggests that at low levels of initial asymmetry, the response to visual feedback is attenuated, and becomes stronger and more linear as the level of asymmetry increases. Although only three participants’ demonstrated moderate to high WBA levels and corresponding improvements with visual feedback, this trend was also observed for participants with WBA ~10%, and is supported by the fit of the smoothing line and strong R^2^ value.

**Figure 3:**
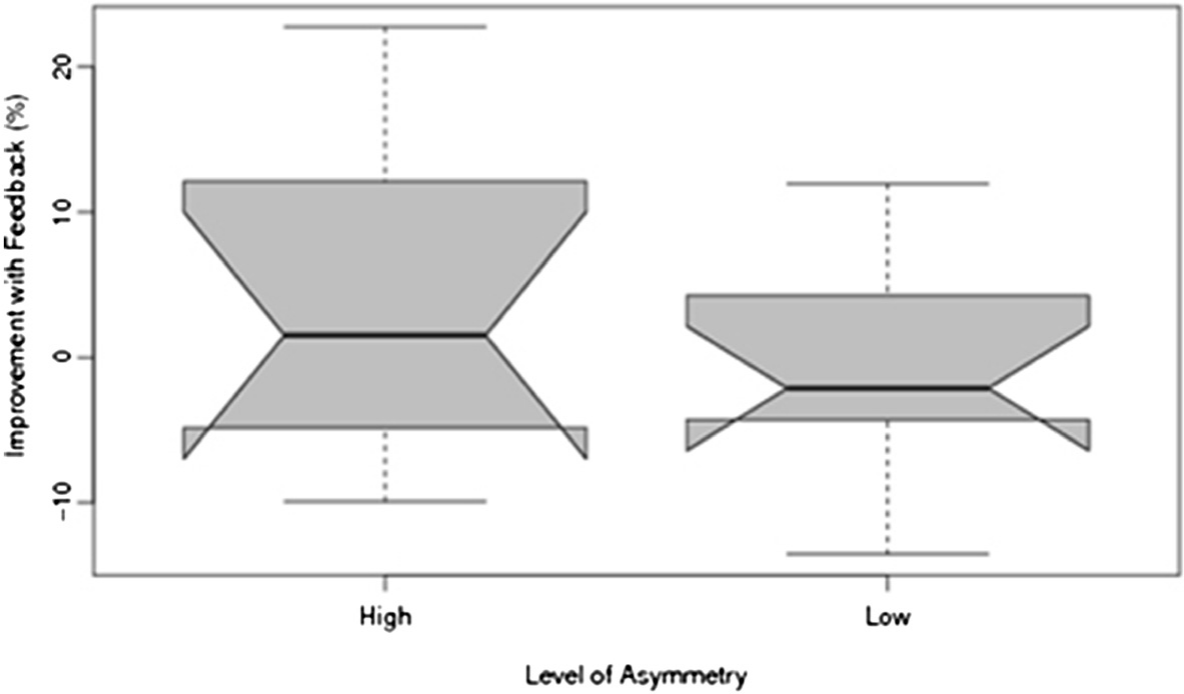
Comparison between high asymmetry and low asymmetry groups in response to visual feedback during the dynamic task.

In inspecting the raw pfSI data for the no visual feedback conditions during the dynamic balance task it became apparent there were two subgroups present – participants who had relatively high asymmetry (highAS; median =23.69) and those who had low asymmetry (lowAS; median = 12.36). The 20 participants were therefore split into two equal sample-size groups (n = 10, cut-off = 19.09) and analysed independently in order to determine if those with differing pfSI scores responded differently to visual feedback. A Mann–Whitney U test confirmed a significant difference (Z = −3.780, *p* < 0.001) between the highAS and lowAS groups without visual feedback. Despite this, no significant change in pfSI was found between the highAS and lowAS groups (Z = −0.756, *p* = 0.450) (Figure 3) when visual feedback was provided.

**Figure 4:**
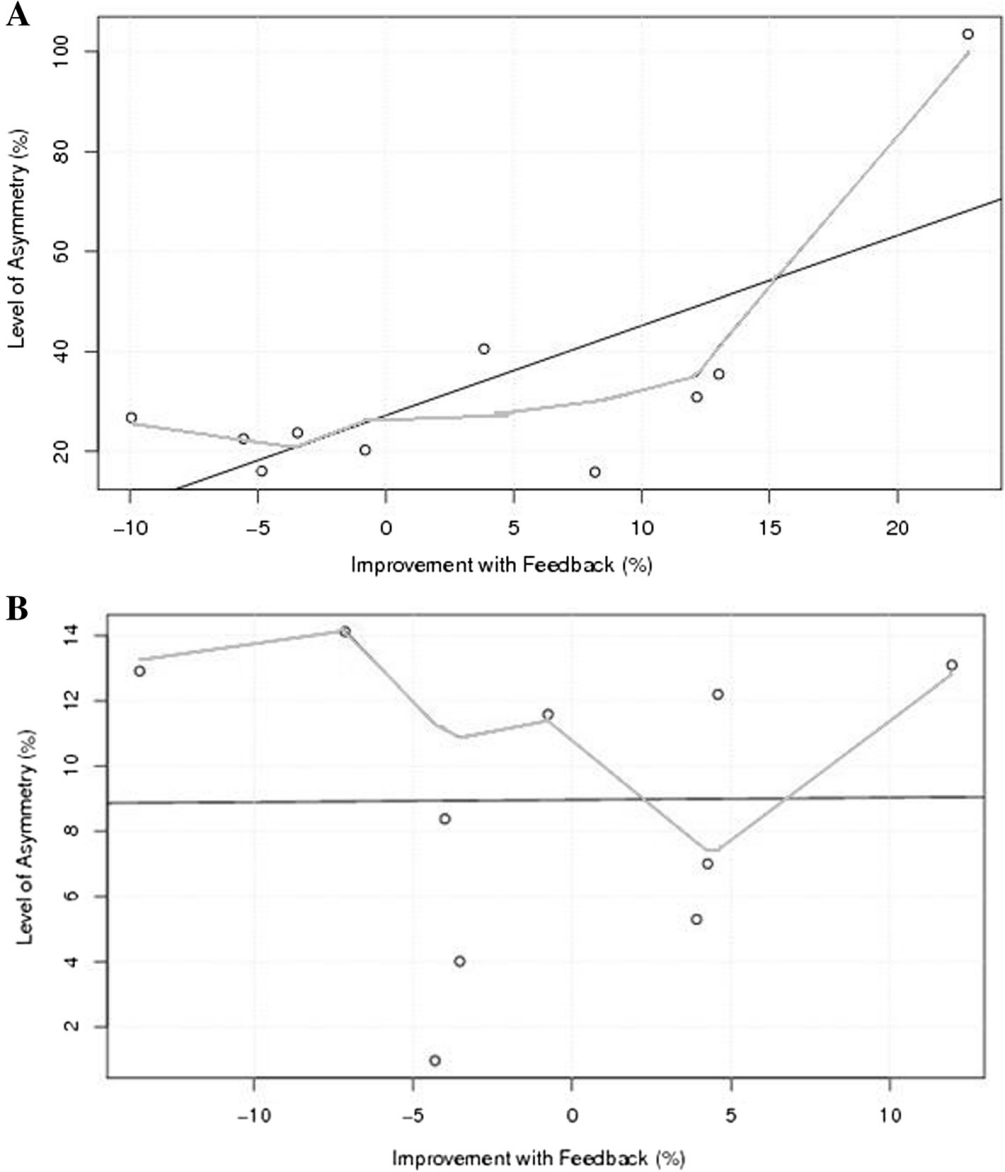
**Relationship between the initial level of asymmetry without visual feedback and the response to visual feedback for the high (A) and low (B) asymmetry groups.** The black, straight line represents the linear fit (high asymmetry: F = 8.535, *p* = 0.019, R^2^ = 0.51; low asymmetry: F < 0.001, p = 0.976, R^2^ < 0.001), the red, irregular line is indicative of the locally weighted scatterplot smoothing line.

Linear regression analyses were performed in order to evaluate the relationship between the initial pfSI without visual feedback and the response to visual feedback for each group in the dynamic task. For the highAS group a significant, positive linear relationship (F = 8.535, *p* = 0.019, R^2^ = 0.51) was observed (see Figure 4a). However, the fluctuating shape of the locally weighted scatterplot smoothing line suggests this response was highly variable between subjects. In contrast, there was no relationship (F < 0.001, p = 0.976, R^2^ < 0.001) observed between these measures in the lowAS group (see Figure 4b).

In regard to the association between the subject’s self-perceived asymmetry and the recorded values, there was no relationship between WBA and VAS for the static balance task (ρ = 0.138, *p* = 0.561) and no relationship between pfSI and VAS for the dynamic balance task (ρ = 0.018, *p* = 0.940) (see Table 2).

**Table 2:** Correlation between the weight-bearing asymmetry and visual analogue scale score for the static balance task, and between the peak force symmetry index and visual analogue scale score for the dynamic balance task

**Comparison**	**Correlation Coefficient**	**Sig. (2-tailed)**
Weight-bearing asymmetry and VAS	0.028	0.907
Symmetry Index and VAS	0.255	0.278

Abbreviation: VAS, visual analog scale.

## Discussion

This is the first study to use two NWBBs to evaluate WBA during static and dynamic balance tasks in people with neurological injuries and to assess customized real-time visual feedback software to improve asymmetry. Participants were from a diverse range of neurological backgrounds, and all were able to complete the tasks with only one familiarisation trial and no adverse events. The results revealed large asymmetries during both the static and dynamic balance tasks when performed without visual feedback. When real-time visual feedback was provided, significant improvements in asymmetry were found in the static task. Inspection of the locally weighted scatterplot smoothing line showed that this response was particularly noticeable as the level of WBA increased, suggesting that the patients who benefitted the most from real-time visual feedback were those who had the most to gain (i.e. those who had higher WBA in the no visual feedback condition).

Other research has also found improvements in standing asymmetry during static balance tasks with the use of visual feedback [26,27]. These studies used force platforms to record limb load, however these items are generally expensive (~$USD20,000 each), require technical expertise, are cumbersome to transport, and as such are mostly confined to a laboratory setting. In contrast, the present study used two inexpensive and portable NWBBs (~$USD100 each) to record individual limb loads, combined with a customized computer software program to provide real-time visual feedback. Therefore, the findings of improved symmetry with visual feedback from the present study suggest that this system may be confidently used in a clinical setting to evaluate and improve measures of WBA during static balance tasks.

Visual feedback was not found to improve symmetry during the dynamic task, however a moderate relationship with performance improvement was observed in those patients with a reasonably high level of initial WBA. Whilst the lack of improvement during the sit-to-stand condition could be due to participant’s either not needing to further correct their WBA or because they are unable due to strength deficits on the affected side, the results from the static trial would suggest that the latter explanation is most likely. That is, the lack of improvement could be due to impairments such as hemiparesis and general muscle weakness of the patient combined with the dynamic nature of the movement. Specifically, during the static trial the patient is able to gradually adjust their weight distribution over many seconds to achieve the desired WBA target; however the standing movement requires this modified control strategy to be implemented rapidly. This may be difficult to achieve for patients who possess neurological deficits, and may be why the response to dynamic feedback in our study was highly variable between subjects. Further research is needed to investigate potential barriers such as weakness and concentration to using visual feedback to correct WBA during dynamic tasks.

The results also revealed little or no relationship between VAS and WBA in the static balance task or between the VAS and the pfSI during the dynamic task when no visual feedback was provided. These findings support those of previous studies [12,15], in that self-perception of WBA during static standing or during a sit to stand task is not related to the actual forces distributed through each limb. This indicates that a participant’s self-perception of how symmetrical they are during balance tasks is inaccurate, and therefore they may not be able to adequately compensate for real asymmetries by simply leaning away from the leg they think is bearing more load. This finding, combined with reported inaccuracies of visual observation of bilateral weight distribution [14], supports the implementation of data acquisition and analysis systems to assess individual limb loading, and reinforces the notion that such a system may be a useful clinical tool for improving a patient’s symmetry levels.

This study had limitations. Visual feedback was only provided in a single acute bout, and as such, the lack of improvement in symmetry during the sit-to-stand condition may be partly due to this short-term nature of the stimulus. Further research should evaluate whether a longer-term program incorporating visual feedback is appropriate for this subject population and leads to functional performance benefits. Additionally, the participants were not formally evaluated to determine whether they possessed the cognitive ability to utilise visual feedback to alter motor performance; however they were judged by the senior treating physiotherapist to be capable of performing the task and responding to the feedback provided. In support of this notion is the strong positive response to the WBA feedback during the static trial, which implies that the participants possessed the cognitive and physical capacity to respond to the visual feedback system utilized in this study. Another potential limitation is the diverse clinical cohort included in this study, with a wide range of neurological conditions represented. This may have been a factor in why the individual subject response to the dynamic WBA feedback was highly variable. Although this is a limitation in the pure research sense, it does represent a typical rehabilitation setting and therefore may be a more ecologically valid assessment of the clinical feasibility of this system.

This study examined the use of an inexpensive WBA assessment and feedback system in a clinical environment with a heterogenous clinical cohort. However, given the aforementioned limitations of this study several issues still need to be addressed. The next research steps may need to focus on 1) How important is correcting WBA during a dynamic task - is the patient’s self-perception of WBA so poor that providing feedback is going to make a difference, and if it does, will this provide longitudinal benefits?, 2) Who may benefit most from visual feedback - is it those with primarily perceptuospatial problems or those with strength deficits?, and 3) Is there a training effect - can the patients learn to improve WBA once they’ve had a series of training sessions? The benefit of similar systems to the one utilized in this study, i.e. low-cost, portable and simple to implement, is that these questions can now be answered in the clinical setting without the time and cost constraints associated with laboratory assessment.

## Conclusion

In conclusion, we found that implementing a portable and inexpensive WBA assessment and feedback system was feasible in a clinical setting representing a diverse range of neurological conditions. We observed high levels of WBA during static and dynamic balance tasks in many of these patients, and found that providing feedback resulted in a rapid positive response during quiet standing. However, during a dynamic task the response to feedback was highly variable, and consequently further research should examine the timeframe required to reduce, and optimal methods of overcoming, high WBA during dynamic tasks.

## Abbreviations

ES: Effect size; highAS: High asymmetry; lowAS: Low asymmetry; NWBB: Nintendo Wii balance boards; pfSI: Peak force symmetry index; VAS: Visual analog score; WBA: Weight-bearing asymmetry.

## Competing interests

The authors have no competing interests to declare. Author RC created and designed the software utilized in this study.

## Authors’ contributions

JF was involved with data acquisition, analysis and interpretation, and writing and reviewing the manuscript. KP contributed towards the conception and organization of the study, and assisted in data acquisition and interpretation, and in writing and reviewing the manuscript. RC developed the software used in the acquisition and analysis of data used in the study, and contributed towards the conception and organization of the study, analysing and interpreting the data and reviewing the manuscript. GA contributed towards the conception and organization of the study and reviewing the manuscript. All authors read and approved the final manuscript.
